# Association of blood group and red blood cell transfusion with the incidence of antepartum, peripartum and postpartum venous thromboembolism

**DOI:** 10.1038/s41598-019-49566-3

**Published:** 2019-09-19

**Authors:** Chen Wang, Isabelle Le Ray, Brian Lee, Agneta Wikman, Marie Reilly

**Affiliations:** 1 Department of Medical Epidemiology and Biostatistics, Karolinska Institutet, Stockholm, Sweden; 2 Centre Hospitalier Régional Universitaire de Strasbourg, Strasbourg, France; 3 Department of Epidemiology and Biostatistics, Drexel University School of Public Health, Philadelphia, USA; 4 Department of Laboratory Medicine, Karolinska Institutet and Department of Clinical Immunology and Transfusion Medicine, Karolinska University Hospital, Stockholm, Sweden

**Keywords:** Preventive medicine, Risk factors

## Abstract

The increased risk of venous thromboembolism (VTE) associated with pregnancy is well-known and prophylaxis guidelines consider a number of risk factors. Although non-O blood group and red blood cell (RBC) transfusion are known to be associated with VTE risk, their contribution to pregnancy-associated VTE has received little attention. This study was conducted in a population-based cohort of 1,000,997 deliveries to women with no prior history of VTE or thrombophilia. The independent contributions of ABO blood type and RBC transfusion to the risks of antepartum, peripartum and postpartum VTE are reported as odds ratios adjusted for risk factors that are considered in current prophylaxis guidelines and other potential confounders. Compared with type O, A and B blood types have higher risk of antepartum and postpartum VTE, with odds ratios between 1.4 and 1.8. Transfusion around delivery has the largest increased risks and a dose-response effect, with adjusted odds ratios from 2.60 (1.71–3.97) for 1–2 units to 3.55 (1.32–9.55) for more than 5 units. ABO blood type and RBC transfusion were found to be independent risk factors for pregnancy-associated VTE. Further research is required to understand the underlying mechanisms and to conduct a risk-benefit assessment of the small volumes of RBCs transfused around delivery.

## Introduction

Venous thromboembolism (VTE) during pregnancy and the postpartum period^1^, including deep vein thrombosis (DVT) and pulmonary embolism (PE), causes a significant global disease burden and is responsible for 13.8% of maternal deaths in developed countries^2^. It is well known that pregnancy is an important risk factor for VTE^3,4^. In Sweden, the VTE risk from 2005 to 2011 among women aged 15–44 years was 42 per 100,000 person years outside pregnancy whereas this number was 5-fold and 10-fold higher during the antepartum and postpartum periods respectively^5^.

Women with previous VTE or thrombophilia are at an increased risk of pregnancy-associated VTE^1^. Other documented risk factors for antepartum VTE include advanced maternal age (>35 years), multiple pregnancy, obesity and smoking, while risk factors for postpartum VTE include Caesarean section, postpartum haemorrhage and stillbirth^3,6^. These and other risk factors are considered in guidelines for preventive interventions for antepartum and postpartum VTE.

Non-O blood group is known to be a risk factor for VTE^7,8^, but there is only scarce evidence reported of its impact on pregnancy-associated VTE^9,10^. Furthermore, there is growing evidence of the role of transfusion in triggering VTE, especially in postsurgical settings^11^ and some authors suggest that transfusion could also be a risk factor for pregnancy-associated VTE^3,6^. The proposed physiopathological mechanism for transfusion-triggered VTE implicates the modulation of inflammatory cascades by transfused RBCs^12^, and a prothrombogenic effect of RBC storage lesions^13^. These mechanisms could also be relevant during pregnancy, which has been shown to alter the cardiovascular physiology^14^. This question is clinically relevant, as transfusion around delivery is frequent due to postpartum haemorrhage, especially when a Caesarean section is performed^15^, and confusion remains about the degree of anaemia that should indicate a transfusion in this setting^16^. A study conducted prior to the introduction of guidelines for thromboprophylaxis in pregnancy in Sweden, found that transfusion (but not post-partum haemorrhage) was an independent risk factor for post-partum VTE^17^ and that there was evidence of a dose-response with number of RBC units received. The main purpose of our work is to take advantage of recent population data sources to investigate whether transfusion is an independent risk factor for pregnancy-associated VTE in the era of thromboprophylaxis, and how such risk depends on the timing of the transfusion, the number of transfused units and the recipient’s blood group.

## Materials and Methods

The Swedish Medical Birth Register MBR^18^, established in 1973 and with 98% coverage from 1987, records numerous maternal characteristics, delivery details, and maternal diagnoses (using International Classification of Diseases codes). At the first antenatal visit, pregnant women in Sweden routinely undergo ABO and RhD blood typing and screening for red blood cell (RBC) antibodies, and this information is recorded in the Scandinavian Donation and Transfusion database (SCANDAT)^19^, which also holds all computerised records of donation and transfusion in Sweden since 1966. The Swedish National Patient Register (NPR) has almost complete coverage of diagnoses and procedures for hospitalised patients from 1987, and outpatient consultations are also recorded since 2001^20^. Diagnoses are recorded using ICD codes whereas procedures are coded using integrated classification of care measures, including classification of surgical measures and non-surgical action codes^21^. The Cause of Death Register (CDR) records date of death with cause of death classified using ICD codes. The unique national registration numbers assigned to all residents in Sweden allows linkage of all data relevant to our study from these sources.

### Defining VTE

The outcome of interest was any ICD code in the NPR or CDR representing venous thrombosis (pulmonary embolism, deep vein thrombosis, pregnancy-associated VTE and other VTE) (Supplemental Table 1). To discriminate between VTE prior to pregnancy, during pregnancy, around delivery and postpartum, we defined VTE in four time intervals: history of VTE - hospital discharge date or outpatient visit date before the estimated conception date; antepartum VTE- hospital stay or outpatient visit after the estimated conception date and before the delivery date; VTE around delivery - hospitalisation including the delivery date with admission date at most 7 days before delivery, or death due to VTE within 7 days of delivery; postpartum VTE - admission after the delivery date and discharge at most 42 days after delivery, or outpatient visit date between the delivery date and 42 days after delivery, or death due to VTE between 7 days and 42 days after delivery.

### Main exposures

Maternal ABO blood group, Rhesus-D status, and transfusion history were extracted from the SCANDAT database. We classified exposure to transfusions into four time intervals: prior history (transfusion date before estimated conception date), during pregnancy (transfusion date between estimated conception date and before the hospitalisation for the delivery), and around delivery (2 days before to 7 days after delivery). Prior history of transfusion and transfusion during pregnancy were defined as binary (Yes/No) variables whereas transfusion around delivery was classified into three levels: 1–2 units, 3–5 units, and more than 5 units. We also identified red blood cell (RBC) transfusions prior to pregnancy, during pregnancy, and around delivery using the same categorisation.

#### Other risk factors

For each pregnancy, information was extracted on maternal age, country of origin, parity, smoking habits, and weight and height at the time of antenatal care registration. We defined advanced maternal age as exceeding age 35 at delivery and grouped mother’s country of origin into three categories (Europe, East or Southeast Asia, and others). Smoking status was dichotomised as ‘smoker’ and ‘non-smoker’. These socio-demographic factors and various pregnancy-related risk factors (multiple pregnancy, mode of delivery, live or stillborn infant) were obtained from the MBR, together with maternal diagnosis of preeclampsia or gestational diabetes (Supplemental Table 1). We classified the mode of delivery as spontaneous vaginal delivery, instrumental vaginal delivery, elective Caesarean section, or emergency Caesarean section.

#### Other medical characteristics

The NPR provided information on surgeries, including orthopaedic procedures and other major surgery (abdominal surgery, cardiovascular surgery such as coronary artery bypass graft surgery, surgery for cancer, and gynaecological surgery). We classified these surgeries as occurring during pregnancy or around delivery using the same definitions as for inpatient records of VTE above. Prior diagnoses (before the pregnancy) of thrombophilia, inflammatory bowel disease (IBD) or other inflammatory or rheumatic diseases was obtained from the ICD codes in the NPR (Supplemental Table 1).

#### Defining the study population

**Figure 1:**
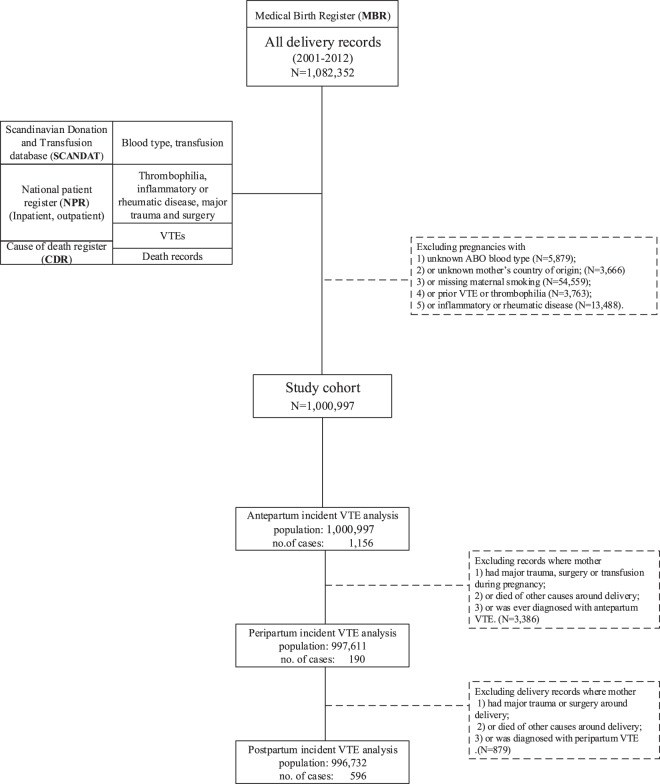
Flowchart depicting sample derivation.

Since many DVT diagnoses will be captured from outpatient records, we defined the start of our study period as 2001, when the Swedish hospitals began registration of outpatient consultations. This also coincides with the introduction of Swedish guidelines for thromboprophylaxis, and thus ensures our study population was not just more recent, but also more homogenous. From the delivery records in the MBR from 2001 to 2012, we extracted all valid delivery records from mothers with a known birth date, and linked these to the Inpatient-, Outpatient-, and Cause of Death registers and to the SCANDAT database to obtain variables of interest (Fig. 1). Since a history of VTE or thrombophilia are known and strong risk factors for recurrence, and may be correlated with a history of transfusion, we focused our investigation on the risk of incident VTE after excluding women with thrombophilia or inflammatory/rheumatic disease. Since we defined the postpartum period from one day up to 42 days (6 weeks) after delivery, we selected pregnancies with estimated conception date from 1st Jan 2001 and at least 6 weeks follow-up after delivery. Excluding women with missing information on blood type and smoking status, the final cohort included 1,000,997 delivery records (Fig. 1). The data were anonymised before being received by the research team. Informed consent was not required for analysis of anonymised population register data. Ethical approval was obtained from the Stockholm Regional Ethics Committee (Diary Number 2012/1133-31/1). We followed the STROBE (Strengthening the Reporting of Observational studies in Epidemiology) guidelines for observational studies.

### Statistical analysis

In preliminary analyses, we compared maternal and pregnancy characteristics, and medical history, between deliveries with and without VTE, using chi-square tests for categorical variables and Student’s t-tests for continuous variables. Separate logistic regression analyses were then conducted for incident VTE during pregnancy, around delivery, and postpartum, to investigate the contribution of RBC transfusion and blood group to the risk of VTE over the three periods. The analysis of incident VTE during pregnancy included all deliveries in the study cohort, whereas incident VTE around delivery or during the postpartum period were analysed after excluding delivery records where the mother was diagnosed with antepartum or peripartum incident VTE, died of other causes, had major trauma or surgery or any blood transfusions during the pregnancy (see Fig. 1). In multivariate logistic regression models, we included known risk factors for VTE, and also some additional variables that are not well-established risk factors but had a plausible time order and some evidence of a crude association with incident VTE (univariate P-value <0.2).

We repeated our multivariate analysis stratified by mode of delivery (vaginal delivery vs. Caesarean section) to investigate potential effect modification of the effect of RBC transfusion.

All data processing and statistical analysis were done using SAS statistical analysis software (Version 9.4, SAS Institute, Inc., Cary, North Carolina).

### Details of ethics approval

The study was approved by the Stockholm Regional Ethical Committee (No. 2008/672-32 and 2012/1:8).

## Results

Of a total of 1,000,997 delivery records, 1950 (0.19%) had a diagnosis of VTE. Overall, there was a higher rate of VTE in women with non-O blood group and lower rates in women who were Asian-born, of younger age or with lower BMI (Table 1). Higher rates of VTE were also observed in women with a prior history of transfusion, with an apparent dose-response relationship for transfusions around delivery. Other pregnancy-associated factors found to be associated with higher rates of VTE were multiple delivery, preeclampsia, Caesarean section, and stillbirth.

**Table 1:** N (%) of pregnancies with incident VTE for each risk factor group, (except maternal age and BMI, where mean ± SD is reported).

	Risk factors		Pregnancy-associated VTE	Population
			N (%)	N
Overall			1950 (0.19)	1000997
Demographic characteristics	Blood group	O	549 (0.14)	382546
		A	1034 (0.23)	441711
		B	274 (0.22)	124337
		AB	93 (0.18)	52403
	Rhesus status	Rhesus positive	1650 (0.19)	856449
		Rhesus negative	300 (0.21)	144548
	Parity	1	852 (0.20)	434683
		≥2	1098 (0.19)	566314
	Mothers origin	Europe	1763 (0.20)	877495
		East and Southeast Asia	22 (0.08)	27834
		Others	165 (0.17)	95668
	Maternal age (mean, SD)		31.4 ± 5.4	30.8 ± 5.2
	Advanced maternal age	>35	462 (0.25)	185672
	Body Mass Index (BMI) mean, SD		25.9 ± 5.1	24.6 ± 4.4
	Obesity	BMI >=30	324 (0.30)	108736
	Mother Smoking status	Smoker	398 (0.23)	176067
Pregnancy details	Multiple delivery		62 (0.43)	14263
	Mode of delivery	Spontaneous vaginal delivery	1315 (0.17)	774314
		Instrumental vaginal delivery	140 (0.19)	74570
		Elective Caesarean section	181 (0.26)	68385
		Emergency Caesarean section	314 (0.38)	83728
	Stillbirth		12 (0.39)	3109
	Gestational diabetes		43 (0.28)	15225
	Preeclampsia		104 (0.35)	29660
	Major trauma or surgery during pregnancy		15 (0.90)	1659
	Major trauma or surgery around delivery		5 (0.71)	704
Transfusion	Prior transfusion		94 (0.31)	30329
	Prior RBC transfusion		86 (0.31)	28049
	Transfusion during pregnancy		6 (1.00)	599
	RBC Transfusion during pregnancy		5 (0.91)	552
	Transfusion around delivery		110 (0.51)	21602
	RBC Transfusion around delivery	1–2 units	62 (0.46)	13496
		3–5 units	34 (0.56)	6124
		>5 units	12 (0.77)	1566

The influence of ABO blood group and RBC transfusion during pregnancy, around delivery, and postpartum, were assessed in multivariate regression models (Table 2). In each of the three periods, there was a similar contribution of blood group A to the risk of incident VTE with an odds ratio ranging from 1.42 to 1.78 when  compared to group O (P < 0.0001). Blood group B was also found to be associated with a higher odds ratio of antepartum and postpartum incident VTE. In contrast, we found no significant association of AB blood group with pregnancy-associated incident VTE in any period. A prior history of RBC transfusion was associated with a 1.41 and 2.29 fold risk of antepartum incident VTE and peripartum incident VTE respectively. In the model assessing the risk in the postpartum period, 1–2 units of RBC transfusion around delivery was found to have a 2.6-fold increase in the odds (95% CI, 1.71–3.97) of having postpartum VTE compared with no RBC transfusions around delivery. This odds ratio increased to 3.55 when the volume of RBC transfusion around delivery was more than 5 units. All these estimates were adjusted for known risk factors and other potential confounders whose contributions are presented in Supplemental Table 2. In stratified analyses of postpartum VTE by mode of delivery the odds ratios for A and B blood types, and RBC transfusions were of similar magnitude for vaginal delivery and Caesarean section (Supplemental Table 3).

**Table 2:** Associations of blood type and RBC transfusion with antepartum, peripartum and postpartum VTE, estimated from a multivariate model.

	Antepartum VTE^*^	Peripartum VTE**	Postpartum VTE***
Population size	1000997	997611	996732
Number of cases	1156	190	596
Adjusted ORs (95% C.I.)			
Blood group			
O	1.0	1.0	1.0
A	**1**.**78 (1**.**55–2**.**04)**	**1**.**48 (1**.**07–2**.**03)**	**1**.**42 (1**.**18–1**.**71)**
B	**1**.**64 (1**.**35–1**.**99)**	0.76 (0.43–1.34)	**1**.**80 (1**.**40–2**.**30)**
AB	1.20 (0.89–1.61)	1.70 (0.95–3.05)	1.17 (0.78–1.74)
Rhesus group			
RhD+	1.0	1.0	1.0
RhD-	0.96 (0.81–1.13)	1.21 (0.83–1.78)	1.17 (0.94–1.45)
Prior RBC transfusion history	**1**.**41 (1**.**05–1**.**89)**	**2**.**29 (1**.**28–4**.**12)**	1.42 (0.95–2.11)
RBC Transfusion around delivery			
None			1.0
1–2 units			**2**.**60 (1**.**71–3**.**97)**
3–5 units			**2**.**98 (1**.**67–5**.**31)**
>5 units			**3**.**55 (1**.**32–9**.**55)**

*The model has been adjusted for calendar year, mother’s country of origin, maternal age, smoking, and multiple gestation. **The model has been restricted to pregnancies with no antepartum VTE, no transfusions or major trauma or surgery during pregnancy, and additionally adjusted for preeclampsia, and gestational diabetes. ***The model has been additionally restricted to pregnancies with no peripartum VTE and no major trauma or surgery around delivery, and additionally adjusted for mode of delivery.

## Discussion

We found indications of increased risk of antepartum and postpartum VTE among women with blood groups A and B. We failed to find an effect for AB blood group or consistent estimates of peripartum risk, but we had limited power in these subgroups. Previous studies have reported risk differences for O and non-O blood groups in hospital patients^22,23^ and in pregnant women^9,24^, effects now understood to be related to ABO-dependent variations in pro-coagulant factor VIII (FVIII) and von Willebrand factor (vWF) levels^8,25^.

A prior history of transfusion was an independent risk factor for antepartum and peripartum VTE. Since the increased risk might be due, at least in part, to the condition that necessitated transfusion, we repeated the analysis for pregnancies of women whose medical history included an indication for transfusion^26^ but the association with history of transfusion persisted, supporting our conclusion that it is an independent risk factor. Transfusion of any number of units of RBCs around delivery was a major independent risk factor for postpartum VTE. A number of studies have reported transfusion as a risk factor for pregnancy-associated VTE^3,17,27,28^. However, one of these studies^3^ did not separate either the exposure (transfusion) or outcome (VTE) into the antepartum, peripartum and postpartum periods, and another study^28^ did not identify the timing of transfusion. By carefully characterising transfusion into different time periods, our study found that there may be both proximal and distal effects of transfusion on VTE risk, with a history of transfusion being associated with higher antepartum risk, which is no longer an independent risk factor postpartum when transfusion around delivery has a large effect. This acute effect of transfusions around delivery could be explained to some extent by a recent study in a North American Register^11^ which suggested that perioperative RBC transfusion may be significantly associated with the development of new or progressive postoperative VTE, independent of several putative confounders. We found the effect to be similar for Caesarean and vaginal delivery, indicating that the delivery process, and not only surgery, can be a trigger. Moreover, we assigned transfusions around delivery into different dose levels and found that any dose contributed to an increased risk of postpartum VTE, consistent with another Swedish study^17^ where a similar adjusted odds ratio (OR 3.3) was reported for 1 to 3 units of RBCs.

A major strength of our study is the prospectively recorded data from population-based registers. Although participants missing information on ABO blood group were excluded, this was only a small proportion of deliveries (5%-8%) and we do not expect it to be a source of bias. Furthermore, our database contained the dates of all transfusions and the type and volume of the products, enabling us to identify and count RBC transfusions, define their time order and investigate a dose-response.

A limitation of the study is our inability to unravel the effect we observed for increasing number of units transfused and the effect of postpartum haemorrhage (a known risk factor for postpartum VTE^17^) since the volume of transfusion will be highly correlated with the severity of bleeding. However, by excluding pregnancies with an ICD code for haemorrhage recorded in the hospital register, we found a significant effect of 1–2 units of RBCs on postpartum VTE risk in pregnancies with no diagnosis of haemorrhage (OR = 2.54; 95% CI: 1.13–5.74), indicating that our finding was not due to the confounding effect of the loss of blood. Another limitation of our study was that the hospital discharge register does not provide the exact dates of disease diagnosis or surgical procedures, so there is potential for misclassification of the timing of VTE. However, half of all VTE diagnoses were from the outpatient register, where the recorded visit date is the diagnosis date. Another limitation was that we had no information on prescriptions for the mothers in our study. While this would help to strengthen our definition of VTE^5^, ICD-codes have been shown to perform well^29^.

For risk of postpartum VTE, transfusion around delivery deserves more attention. We confirmed RBC transfusion, even of small volume, to be an independent risk factor. Since treatment or prevention of anaemia accounts for the majority of transfusions to obstetric patients, the small-volume transfusions may be avoidable, as randomised controlled trials have demonstrated reassuring results for alternative treatments, such as iron supplementation^30^. Our findings can contribute to the debate concerning efficacy of peripartum transfusion and provide evidence for updating guidelines for VTE prophylaxis in pregnant women.

## Supplementary information

Supplementary Tables

## Data Availability

The individual-level data can only be shared in the context of an agreed collaboration and subject to a data-sharing agreement to ensure security of personal data.
