# Supplementary material for: Association of blood group and red blood cell transfusion with the incidence of antepartum, peripartum and postpartum venous thromboembolism
Source: Sci Rep. 2019 Sep 19;9:13535. doi: 10.1038/s41598-019-49566-3 (PMC6753067; doi:10.1038/s41598-019-49566-3)
Supplement: Supplementary file 1 — Supplementary Tables [file 41598_2019_49566_MOESM1_ESM.pdf]

Supplemental Material for manuscript:

Association of blood group and red blood cell transfusion with the incidence of antepartum, peripartum and postpartum venous thromboembolism

by

Chen Wang, Isabelle Le Ray, Brian Lee, Agneta Wikman, Marie Reilly

Supplemental Table 1. *International Classification of Disease (ICD) codes used to define medical diagnoses during the study period: ICD-9 classification for1987-1996 and ICD-10 from 1997.*

	ICD-9	ICD-10
**VTE**		
Pulmonary embolism	415	I26
Deep vein thrombosis	451B,	I801, I802
Pregnancy-related venous thrombosis	671C,671D,671E,	O087, O223, O871, O882
Other venous thrombosis	453A, 453C, 453D	I81, I820, I822, I823, I636, I676
**Other medical complications**		
Thrombophilia	289.81	D685, D686
Inflammatory bowel disease	5550,5551,5552,5559,556	K50, K51
Other inflammatory or rheumatoid disease	5810,5811,5812,5813,7100,7101,7102,7103,7104,7108,7140,7142,7148,7149	N04, M05, M06, M07, M13, M03
**Pregnancy-associated complications***		
Preeclampsia		O14, O15
Gestational diabetes		O24

*Study cohort consists of pregnancies from 2001-2012 when ICD-10 classification was in use.

Supplemental Table 2: *Associations of risk factors with antepartum, peripartum and postpartum VTE, estimated from multivariate models.*

	Antepartum VTE*	Peripartum VTE**	Postpartum VTE***
Population size	1000997	997611	996732
Number of cases	1156	190	596
		Adjusted ORs (95% C.I.)	
Blood group			
O	1.0	1.0	1.0
A	**1.78(1.55-2.04)**	**1.48(1.07-2.03)**	**1.42(1.18-1.71)**
B	**1.64(1.35-1.99)**	0.76(0.43-1.34)	**1.80(1.40-2.30)**
AB	1.20(0.89-1.61)	1.70(0.95-3.05)	1.17(0.78-1.74)
Rhesus group			
RhD+	1.0	1.0	1.0
RhD-	0.96(0.81-1.13)	1.21(0.83-1.78)	1.17(0.94-1.45)
Advanced maternal age	**1.27(1.10-1.45)**	**1.51(1.09-2.08)**	**1.42(1.18-1.70)**
Multiple gestations	**2.01(1.42-2.82)**	**4.20(2.37-7.42)**	1.11 (0.67-1.84)
Preeclampsia		**2.75(1.65-4.56)**	**2.29(1.71-3.07)**
Gestational diabetes		1.40(0.57-3.43)	0.79(0.42-1.47)
Delivery mode			
Spontaneous vaginal delivery			1.0
Instrumental vaginal delivery			0.93(0.66-1.30)
Elective caesarean section			**1.73(1.33-2.26)**
Emergency caesarean section			**2.39(1.93-2.96)**
Prior RBC transfusion history	**1.41(1.05-1.89)**	**2.29(1.28-4.12)**	1.42(0.95-2.11)
RBC Transfusion around delivery			
None (0 unit)			1.0
1-2 units			**2.60(1.71-3.97)**
3-5 units			**2.98(1.67-5.31)**
>5 units			**3.55(1.32-9.55)**

*The model has also been adjusted for calendar year, mother's country of origin and smoking status. **The model has been restricted to pregnancies with no antepartum VTE, no transfusions or major trauma or surgery during pregnancy. *** The model has been additionally restricted to pregnancies with no peripartum VTE and no major trauma or surgery around delivery.

Supplemental Table 3: *Analysis of postpartum incident VTE, stratified by mode of delivery.*

	Postpartum incident VTE	
	**Vaginal delivery**	**Caesarean section**
Population size	845869	150863
Number of cases	420	176
	Adjusted* ORs (95% C.I.)	
Blood group		
O	1.0	1.0
A	**1.44(1.15-1.80)**	1.36(0.97-1.90)
B	**1.95(1.46-2.60)**	1.47(0.92-2.34)
AB	1.34(0.85-2.10)	0.79(0.34-1.85)
Rhesus group		
RhD+	1.0	1.0
RhD-	1.22(0.95-1.57)	1.06(0.70-1.60)
Prior RBC transfusion history	**1.69(1.06-2.68)**	0.92(0.43-1.97)
RBC Transfusion around delivery		
None (0 unit)	1.0	1.0
1-2 units	**2.71(1.55-4.72)**	**2.54(1.34-4.85)**
3-5 units	**3.47(1.72-7.03)**	2.35(0.87-6.40)
>5 units	3.82(0.95-15.42)	3.35(0.82-13.66)

*The model has been adjusted for calendar year, mother’s country of origin, maternal age, smoking, multiple gestation, preeclampsia, and gestational diabetes.
